# Annotations

**Published:** 1902-07-12

**Authors:** 


### ANNOTATIONS.

### Physicians, Surgeons, and Appendicitis.

Surgery is nowadays so greatly in the ascendant and poor medicine is so often made to feel that her days are o'er, except so far as she may act as jackal to her exacting rival, that it is with a certain amount of pleasure, not unmixed with amusement, that we find the physicians insisting that when the surgeons say that appendicitis "less than twenty years ago" was "practically unrecognised," they ought to say unrecognised "by surgeons." Dr. Pye-Smith points out that, although it may be true enough that this important disease was unrecognised by surgeons and found no place in the manuals of surgery, it was not 20 but more than 40 years ago, recognised by physicians and found an accepted place in manuals of medicine. He says that in the *Elements of the Practice of Medicine*, published in 1836, Addison after describing the pain, swelling, and tenderness, with frequently consequent suppuration, and occasionally general and fatal peritonitis, wrote as follows :—

> From numerous dissections it is proved that the fæcal abscess thus formed in the right iliac region arises in the great majority of instances from disease set up in the appendix cæci. This organ is very subject to inflammation, ulceration, and even to gangrene.

This view he believes was generally adopted by physicans long before 1875, when it was stated as an undoubted fact by Wilks and Moxon. They gave the condition its proper name of "typhlitis, or inflammation of the cæcum and appendix." Dr. Bristowe also used the word typhlitis in the same sense in the fourth volume of Reynolds's "System" published in 1871. Surgeons may have taken no heed, but evidently the physicians knew about the disease a long time ago. Sir Samuel Wilks also writes in the same strain, saying that the disease has been known and recognised ever since he entered the profession more than fifty years ago, and that the only novelty at the present time seems to be the use of the word "appendicitis," an uncouth and improper expression. The truth of the matter would seem to be that in this as in many other instances, the pathological knowledge which we already possessed failed of its full fruition until such advances had been made in surgery as made it possible to open the abdominal cavity with confidence and safety.

### The "Flatites."

An appeal has been issued on behalf of the Grosvenor Hospital for Women and Children, Vincent Square, Westminster. It is pointed out that the work of the hospital is almost entirely surgical, that many serious operations are performed within its walls, and that the comparative privacy of the wards, owing to the absence of students, is greatly appreciated by the patients. Much stress is laid upon the poverty of the district in the midst of which it is situated, and no one who knows the neighbourhood can have any doubt about the impossibility of many of its inhabitants obtaining proper surgical assistance except within the walls of a well-appointed hospital. While, however, we willingly give prominence to the claims of this charity we would point out that within almost a stone's throw there is a mine of irresponsible wealth which as we often suspect is not half as fully exploited as it deserves to be—we mean the "Flatites." There is probably no class in the community which in proportion to its wealth gives so little to the general good as the dwellers in flats. Within a few hundred yards of Victoria Street there must be many hundreds of people living in flats at rents of from £200 to £500 a year, who, if they lived in houses of equivalent importance would almost certainly be subscribers to all sorts of local charities. But the "Flatites" escape; many are not called upon at all, and many, when asked, have an airy way of saying that the flat is only a temporary affair, and that they have calls upon them at their "place" in the country. Which is all very fine; but they fill up the district and enjoy the best that London offers, and, except so far as rent is concerned, they do this at a very moderate expense, and they ought to pay up to local charities. We commend this to some persistent and oily-tongued collector. This particular hospital has especial claims upon the "flat" district which lies so close to its doors, in that it takes in a certain number of paying patients at a very moderate weekly sum. Flats are very well to live in, but they offer no convenience for illness, and all the good "Flatites" down Victoria Street ought to be extremely glad to support a hospital at their very doors in which a private room can be had for so moderate a sum as a couple of guineas a week.

### Intra-Tracheal Injections in Phthisis.

A good deal has recently been heard about a mode of treating pulmonary phthisis by the intra-tracheal injection of various drugs. Dr. Colin Campbell has more particularly advocated this method, and has surprised many people, who did not know how tolerant the trachea is if properly dealt with, by his statements as to the quantity of material which he is able thus to inject into passages whose normal function seems so opposed to such treatment. The cases which he reports, however, are good, and must be accepted with the respect due to a man who, as he has recently stated, has administered upwards of 219,000 separate injections. One is staggered by such numbers. Still there they are, and they must be taken as part of the evidence on which this treatment must stand or fall. There are some sceptical and hardheaded persons, no doubt, who will say that such figures add no force to, and in fact rather detract from, the value of the rest of the evidence, and we can well imagine that certain sober medical practitioners who are able to appreciate the labour involved in the performance of one particular operation, or manœuvre, or whatever one likes to call it, such an enormous number of times, will shake their heads. All we have to do is to express our admiration of the perseverance of a man who amidst the various duties which have devolved upon him in the many important posts which he has held—such as those of workhouse doctor, public vaccinator, and factory surgeon—should have found time and energy for the enormous labour involved in such an undertaking—219,000 injections! The thing is prodigious! Fancy a man for fifteen years of his life doing this operation on an average forty times a day for every day in every year. What energy! what enthusiasm! what untiring industry!

